# Deciphering New Molecular Mechanisms of Mast Cell Activation

**DOI:** 10.3389/fimmu.2013.00100

**Published:** 2013-04-25

**Authors:** Ulrich Blank, Marc Benhamou

**Affiliations:** ^1^UMRS 699, INSERMParis, France; ^2^Laboratoire d'excellence, INFLAMEX, Université Paris-Diderot, Sorbonne Paris CiteParis, France

Mast cell were initially studied mostly for their implication in type I hypersensitivity and allergies (Blank and Rivera, 2004; Galli et al., 2008b). However, research over the last 20 years has made clear that they do more than just causing a runny nose or asthma attacks. In fact, they have emerged as prime actors of immune and inflammatory responses. As such they sense their environment via numerous cell surface expressed receptors to orchestrate an appropriate immune and inflammatory response. Work from many laboratories has made clear that mast cells participate in the restoration of tissue homeostasis following an offensive event contributing for example to the elimination of infectious agents as well as to tissue repair and remodeling responses (Marshall, 2004; Galli et al., 2005; Blank et al., 2007; Abraham and St John, 2010; Beghdadi et al., 2011).

Mast cells are tissue-localized effectors of hematopoietic origin. They are particularly prominent in tissues that are in contact with the external environment such as the skin, the gastrointestinal tract, or the airways. Yet, they are present also in many other tissues of an organism and often their numbers increase upon an inflammatory reaction. They can be found at strategic locations close to blood capillaries in order to rapidly communicate to other hematopoietic effectors to enter into action. They are also found in contact with nerve terminals allowing these cells, via released mediators, to communicate to the brain the presence of environmental dangers or reversely to respond to signals emanating from the brain, thus providing a relay between the central nervous system and the immune system (Theoharides et al., 2012).

**Figure 1:**
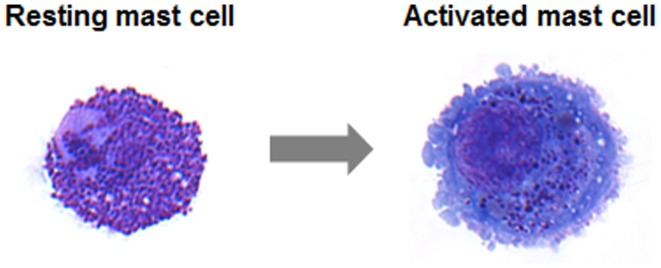
**May–Grünwald/Giemsa stain of a resting human intestinal mast cell and a mast cell following activation induced degranulation**. Note the increase in size and loss of granule staining (Figure from Lorentz et al., 2012).

One of the prime characteristics of mast cells is to release rapidly within a few minutes upon activation a whole set of inflammatory products by cellular degranulation (Figure 1) including histamine and proteases, proteoglycans, lysosomal enzymes, etc. (Blank and Rivera, 2004). As a consequence, blood vessels dilate thus increasing the blood flow in the offended area. Moreover, under the effect of released histamine, vessels become permeable allowing the influx of other inflammatory cells and other inflammatory products (such as for example immunoglobulins and complement) into tissues to mount an inflammatory response against the insult. In a more delayed response mast cells also produce lipid mediators such as prostaglandins and leukotrienes, which enhance some of the initial tissue responses and promote supplementary responses such as increased temperature (fever), smooth muscle contraction, etc. This is followed by a third wave of mediators, which correspond to various cytokines and chemokines allowing to enhance the flow of other inflammatory and immune cells into tissue, but also to start to regulate and coordinate this inflammatory response. Interestingly, the array of mediators released may differ depending on the type of stimulus. Thus, stimulation of Toll-like receptor 4 (TLR4) by bacterial products such as LPS usually does not promote the first wave of degranulation, but rather leads to an enhanced production of cytokines and chemokines (Leal-Berumen et al., 1994). Therefore, in addition to releasing a whole set of inflammatory mediators, mast cells can initiate different actions allowing these cells to fine-tune the inflammatory response. Indeed, evidence has emerged that mast cells, depending on the type of stimulus initiate different actions that can have opposite pro- or anti-inflammatory consequences (Metz et al., 2007; Galli et al., 2008a; Beghdadi et al., 2011). Interestingly, in some cases this may depend on the strength of the stimulus or on its timing in the course of an inflammatory reaction (Beghdadi et al., 2011).

While in the past many reviews have focused on the capacity of these cells to respond to IgE-mediated activation, the focus of this ebook of *Frontiers in Innate Immunology* is devoted substantially to emphasize other types of stimuli for mast cells and the molecular mechanisms involved, as well as to highlight new types of responses by these cells. Thus, several reviews of this ebook describe new types of stimuli for these cells involving chemokines (Halova et al., 2012), sex hormones (Zierau et al., 2012), tetraspanins (Koberle et al., 2012), TRP channels (Freichel et al., 2012), TLRs (Sandig and Bulfone-Paus, 2012). Another article provides a general description of novel identified receptors (Migalovich-Sheikhet et al., 2012) that may act also as inhibitory receptors of an inflammatory reaction. Some contributions deal with the regulation of mast cell activation involving for example cytoskeletal elements (Draber et al., 2012), ion channels or tetraspanins (Freichel et al., 2012; Koberle et al., 2012), or the mechanism involved in secretory granule fusion or the crosstalk between different cell surface receptors (Lorentz et al., 2012; Migalovich-Sheikhet et al., 2012). Another point discussed is the implication of these stimuli in new types of biological responses mediated by mast cells. This includes for example a description of how female sex hormones can influence allergic asthma, how these hormones participate in mast cell uterine functions (Zierau et al., 2012) and how mast cells can interfere with reproductive processes (Woidacki et al., 2013). Another chapter analyses the interaction of mast cells with other immune cells discussing the receptors and mediators involved in these new types of connections (Gri et al., 2012).

Thus, while in the past mast cells have been often discussed with respect to their participation in allergic type of responses this collection of specific chapters aims to stress that these cells are in fact versatile inflammatory effectors with multiple functions in the organism. Indeed, cells resembling mast cells that contain histamine and proteoglycans such as heparin have already been recognized in tunicates (Cavalcante et al., 2002), which are amongst the first multicellular organism preceding vertebrates. This is well before the appearance of IgE, making clear that these cells probably are part of an ancient immune surveillance system allowing the organism to defend itself against tissue damage and organize physiological responses within tissues.
